# A firm-level dataset for analyzing entry, exit, employment and R&D expenditures in the UK: 1997–2012

**DOI:** 10.1016/j.dib.2016.05.028

**Published:** 2016-05-21

**Authors:** Mehmet Ugur, Eshref Trushin, Edna Solomon

**Affiliations:** a University of Greenwich Business School, United Kingdom; b Durham University Business School, United Kingdom

**Keywords:** R&D, Innovation, Firm dynamics, Survival analysis

## Abstract

This data article is related to the research article entitled “Inverted-U relationship between R&D intensity and survival: Evidence on scale and complementarity effects in UK data” (Ugur et al., In press) [1]. It describes the trends in R&D expenditures, employment of R&D personnel and firm entry and exit rates in the UK from 1998 to 2012. We also provide statistics on net employment creation and net R&D investments due to firm entry and exits. In addition, we compute the correlation coefficients between entry and exit rates at the two digit industry level so as to examine whether the correlations are contemporaneous or inter-temporal. Finally, we provide information about the underlying dataset to which secure access is available through *UK Data Service Archive* 7716 at http://dx.doi.org/10.5255/UKDA-SN-7716-1.

**Specifications Table**

**Table:** 

Subject area	*Economics*
More specific subject area	*Survival analysis, R&D*
Type of data	*Tables and graphs*
How data was acquired	*Data was acquired by merging the ONS datasets on Business Expenditure and Research Database (BERD) and the Business Structure Database (BSD). These databases are available from the UK Data Service repository (https://www.ukdataservice.ac.uk/)*
Data format	*Aggregated, analyzed*
Experimental factors	*We make use of observational data based on annual surveys.*
	*Our sample was extracted by merging information from the BSD and BERD databases using the STATA software. Sample construction involved various consistency checks.*
	*The final dataset we make available is a long panel at the firm level from 1997 to 2012.*
Experimental features	*Data on employment, R&D expenditures, entry and exit rates is aggregated from reporting unit to enterprise unit level.*
Data source location	*United Kingdom*
Data accessibility	*Data are within this article. The underlying dataset is available through secure access via UK Data Service Archive SN7716 at:* http://dx.doi.org/10.5255/UKDA-SN-7716-1 [2]

**Value of the data**

• Fig. 1, Fig. 2 on R&D expenditure and employment of R&D personnel, together with the underlying dataset from 1997–2012, could inform further research on determinants of R&D expenditures and employment of R&D personnel.
• Annual statistics on entry and exit rates in Table 1 highlight the implications of firm dynamics (entry and exit rates) for job creation, job destruction and net R&D expenditure. Furthermore, the underlying dataset can stimulate further research on firm dynamics, labor reallocation and productivity.
• The correlation table between entry and exit rates in Table 2 can inform further research on the lack of sorting out effects in firm dynamics in the UK.
• The link to the underlying dataset provides researchers with consistent and reliable microdata on UK firms from 1997 to 2012. The dataset has significant potential for future research in areas such as: (a) size distribution of firms; (b) firm diversity and survival; (c) geographical spillovers of R&D; and (d) job creation versus job destruction during the crisis and post-crisis periods.

## 1 Data

**Fig. 1:**
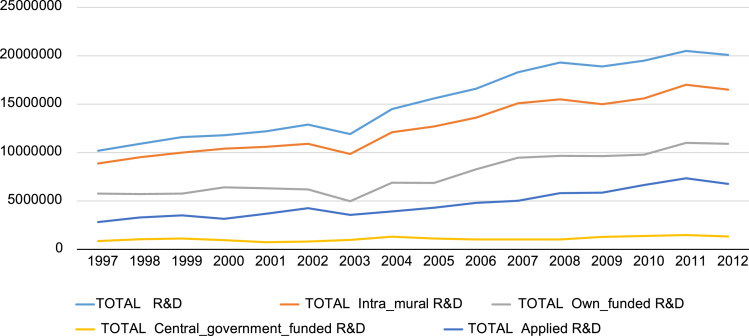
R&D expenditure by type: 1997–2012 (£ ‘000).

**Fig. 2:**
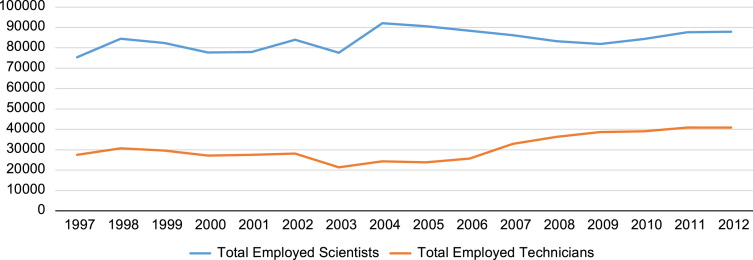
Employed scientists and technicians, total: 1997–2012.

In this article, first we present two graphs depicting the trends in R&D expenditure by type (Fig. 1) and by R&D personnel (Fig. 2), drawing on the panel dataset we constructed from two Office for National Statistics (ONS) databases for the period 1997–2012. These are followed by Table 1 on annual entry and exit rates, net balances of employment and net balances of R&D investment, using data for 37,930 UK firms from 1998 to 2012. Table 2 follows with correlations between firm entry and exit rates at 3-digit industry level - with and without correction for industry fixed effects.

## 2 Experimental design, materials and methods

### 2.1 Dataset: sources and indicative content

Our dataset was obtained by merging the Business Expenditure on Research and Development (BERD) [3] with the Business Structure Database (BSD) [4]. The BERD database is an annual survey of firms with information on research and development. The BSD database is an annual snapshot of the *Inter-Departmental Business Register* (IDBR) – a live register of all UK firms registered for value-added tax (VAT) and/or Pay-as-You-Earn (PAYE) tax purposes. We merged the two datasets using the unique enterprise identifier. The merged dataset contains 37,930 firms with 185,094 firm/year observations after excluding firms with incorrect birth dates.

The merged dataset [2] contains demographic firm information such as births and deaths as well as employment, turnover, R&D measures, SIC codes, etc. We ensured that all key variables were cleaned and consistent across years. Finally, the dataset was augmented with derived variables such as SIC-2007 consistent industry classification codes, UK versus foreign ownership codes, consistent output deflators, Pavitt classes and Herfindahl index at the 3 digit level. Further information on the merging and cleaning process is available in [1].

### 2.2 R&D investment and personnel

Total R&D expenditures from 1997 to 2012 is presented in Fig. 1. R&D expenditures are also broken down into Intramural R&D, Applied R&D and two key sources of R&D funding (own-funded R&D and R&D funded by the UK Central government). This is followed by Fig. 2, which depicts the trend in R&D personnel (scientists and technicians employed with the purpose of conducting R&D) from 1997 to 2012.

### 2.3 Entry and exit by year and industry

Annual entry and exit rates, together with associated changes in employment and total R&D expenditures are given in Table 1. The table summarizes the entry and exit rates by year and over the whole period from 1998–2012. It also summarizes the net balances of employment and R&D expenditures after taking into account the values associated with new entrants and exiting firms. The annual figures allow for comparing entry and exit rates and employment and R&D expenditure balances before, during and after the financial crisis of 2007–2009.

In Table 2, we present the correlations between entry and exit rates at the industry level, with and without correction for industry fixed effects (See [5], [6]). Uncorrected entry and exit rates by year and 2-digit industry are calculated in accordance with Eq. (1).

(1) $${\mathrm{NR}}_{\mathrm{it}}=N_{\mathrm{it}}/F_{\mathrm{it}-1}\;\mathrm{and}\;{\mathrm{XR}}_{\mathrm{it}}=X_{\mathrm{it}}/F_{\mathrm{it}-1}$$

where ${\mathrm{NR}}_{\mathrm{it}}$ and ${\mathrm{XR}}_{\mathrm{it}}$ are entry and exit rates in industry *i* in year *t*; $N_{\mathrm{it}}$ and $X_{\mathrm{it}}$ are numbers of entrants and exiters in industry *i* in year *t*; and $F_{\mathrm{it}-1}$ is the total number of firms in industry *i* in the previous year. Uncorrected entry and exit rates enable us to verify if correlations are contemporaneous or inter-temporal, and whether the correlations reflect industry-specific fixed effects due to technological conditions. We also calculated entry and exit rates corrected for fixed industry effects. The latter allows for verifying the existence of a ‘sorting effect’ and is calculated in accordance with Eq. 2, where ${\mathrm{NR}}_{\mathrm{it}}^{*}$ and ${\mathrm{NR}}_{\mathrm{it}}^{*}$ are demeaned entry and exit rates; and ${\bar{\mathrm{NR}}}_{i}$ and ${\bar{\mathrm{XR}}}_{i}$ are average entry and exit rates by industry.

(2) $${\mathrm{NR}}_{\mathrm{it}}^{*}={\mathrm{NR}}_{\mathrm{it}}-{\bar{\mathrm{NR}}}_{i}\;\mathrm{and}\;{\mathrm{XR}}_{\mathrm{it}}^{*}={\mathrm{XR}}_{\mathrm{it}}-{\bar{\mathrm{XR}}}_{i}$$

When industry-fixed effects are not corrected for, we can verify whether correlations are contemporaneous or inter-temporal, irrespective of industry-specific technological conditions. The evidence is in line with earlier findings from UK and US data [5], [6]. It indicates that periods of high (low) entry are also periods of high (low) exit – irrespective of differences in industry-specific technological conditions. However, entry and exit rates are not correlated inter-temporally. Again this is in line with existing findings and indicates that periods of high (low) entry are not followed by periods or high (low) exit.

When corrected for industry-specific fixed effects, the correlations between entry and exit rates indicate whether a ‘sorting effect’ is at work. The latter arises if the marginal entrant in the period of above-average entry is of worse quality and, hence, increases the exit rate in the subsequent period. The findings in the off-diagonal cells of the right panel indicate that a sorting effect is not at work in UK data. The findings in the diagonal cells indicate contemporaneous correlations between entry and exit rates are not driven by industry-specific fixed effects. This is in line with evidence from UK data in [5], but in contrast to evidence from US data in [6], where a sorting effect exists.

## Figures and Tables

**Table 1:** Entry, exit rates and associated balances in employment and total R&D expenditures.

Year	Entry rate	Exit rate	Employment	R&D balance
	(%)	(%)	(Balance, ‘000)	(Balance, £ Mn)
1998	6.5	0.7	30.8	1355.6
1999	4.7	1.2	7.2	−177.2
2000	5.0	1.7	46.4	645.9
2001	5.1	1.2	114.5	3063.2
2002	5.7	1.2	12.9	1433.6
2003	5.3	1.9	35.1	8525.2
2004	4.4	1.7	−77.1	112.2
2005	4.2	1.5	26.8	−97.2
2006	2.9	1.8	5.2	152.0
2007	4.7	2.7	18.1	38.0
2008	5.1	4.6	2.0	406.6
2009	1.7	2.0	−7.0	58.1
2010	2.1	2.3	−11.3	−91.2
2011	1.5	2.2	−6.2	27.8
2012	0.8	2.2	1.3	−131.3
Overall	4.0	1.9	198.8	15,321.3

**Table 2:** Correlation between entry and exit rates by 2-digit industry and year.

**Ent. Rate Ext. rate**	**Uncorrected for fixed effects**				**Corrected for fixed effect**			
	1998–2002	2003–2007	2008–2012	1998–2012	1998–2002	2003–2007	2008–2012	1998–2012
1998–2002	**0.020**	0.07	0.15	–	−**0.005**	0.02	0.15	–
2003–2007	−0.06	**0.73**	−0.03	–	−0.04	**0.72**	−0.13	–
2008–2012	0.17	−0.05	**0.89**	–	0.23	−0.20	**0.86**	–
1998–2012	–	–	–	**0.63**	–	–	–	**0.63**
