# Supplementary material for: A firm-level dataset for analyzing entry, exit, employment and R&D expenditures in the UK: 1997–2012
Source: Data Brief. 2016 May 21;8:153–7. doi: 10.1016/j.dib.2016.05.028 (PMC4889978; doi:10.1016/j.dib.2016.05.028)
Supplement: Supplementary file 1 — Supplementary material [file mmc1.docx]

**Conflicts of Interest Statement**

Manuscript title: *A firm-level dataset for analyzing entry, exit, employment and R&D expenditures in the UK: 1997-2012*

The authors whose names are listed immediately below certify that they have NO affiliations with or involvement in any organization or entity with any financial interest (such as honoraria; educational grants; participation in speakers’ bureaus; membership, employment, consultancies, stock ownership, or other equity interest; and expert testimony or patent-licensing arrangements), or non-financial interest (such as personal or professional relationships, affiliations, knowledge or beliefs) in the subject matter or materials discussed in this manuscript.

Author names:

Mehmet Ugur

Eshref Trushin

Edna Solomon
